# Two-year, prospective, multicenter study of the use of dexamethasone intravitreal implant for treatment of macular edema secondary to retinal vein occlusion in the clinical setting in France

**DOI:** 10.1007/s00417-016-3394-y

**Published:** 2016-06-11

**Authors:** Jean-François Korobelnik, Laurent Kodjikian, Cécile Delcourt, Vincent Gualino, Richard Leaback, Sybil Pinchinat, Marie-Eve Velard

**Affiliations:** 1 Service d’Ophtalmologie, CHU de Bordeaux, Bordeaux, France; 2 Univ. Bordeaux, ISPED, F-33000 Bordeaux, France; 3 Inserm, U1219 - Bordeaux Population Health Research Center, F-33000 Bordeaux, France; 4 La Croix-Rousse Hospital, University Hospital of Lyon, Lyon, France; 5 Clinique Honoré Cave, Montauban, France; 6 Allergan plc, Marlow, UK; 7 Biostatem, Castries, France; 8 Allergan plc, Courbevoie, France

**Keywords:** Dexamethasone, Intravitreal, Macular edema, Observational, Prospective, Retinal vein occlusion

## Abstract

**Purpose:**

To evaluate patterns of use and long-term efficacy and safety of dexamethasone intravitreal implant (DEX implant) in the treatment of macular edema secondary to branch or central retinal vein occlusion (BRVO, CRVO) in French clinical practice.

**Methods:**

A 24-month, prospective, multicenter, longitudinal, observational study (LOUVRE) conducted at 48 randomly selected sites in metropolitan France enrolled consecutive adult patients with macular edema following retinal vein occlusion (RVO) who were treated with DEX implant at baseline. Re-treatment with DEX implant and use of other RVO treatments was at the physician’s discretion. The primary endpoint was the change in best-corrected visual acuity (BCVA) from baseline to month 6. Secondary endpoints included change in BCVA, intraocular pressure (IOP), adverse events, and RVO treatments administered through month 24.

**Results:**

The analysis population of 375 patients (53.9 % BRVO, 46.1 % CRVO) received a mean of 2.6 DEX implant injections over 2 years; mean time between injections was 6.6 months. Mean (SD) change in BCVA from baseline was 5.1 (19.0) letters at month 6 (*p* < 0.001) and 4.6 (22.3) letters at month 24 (*p* < 0.001). During the study, 208 patients (55.5 %) received treatment other than DEX implant for RVO, usually laser or ranibizumab therapy, with first use of other therapy occurring at a mean of 8.7 months. Mean change from baseline BCVA at month 6 was 5.5 letters (*p* < 0.001, *N* = 254) in patients who had received only DEX implant and 4.2 letters (*p* = 0.006, *N* = 121) in patients who had received additional other RVO treatment during the first 6 months. At month 24, mean change from baseline BCVA was +20.7 letters in patients treated with a single DEX implant only (*p* < 0.001), +4.9 letters in patients treated with ≥2 DEX implants only (*p* = 0.029), and +2.3 letters in patients treated with DEX implant and other RVO treatment (*p* = 0.143). The most common adverse events (incidence) were cataract progression (39.7 %) and increased IOP (34.4 %). No glaucoma incisional surgeries were required.

**Conclusions:**

Efficacy and safety of DEX implant in the treatment of RVO-associated macular edema were demonstrated in the French clinical setting. Patients who switched from DEX implant to other RVO treatments did not have improved outcomes.

The study is registered at ClinicalTrials.gov with the identifier NCT01618266.

## Introduction

Retinal vein occlusion (RVO), typically characterized as branch or central RVO (BRVO, CRVO), is the second most common sight-threatening ophthalmic vascular disease [1] affecting an estimated 16 million individuals worldwide [2]. Macular edema (ME), a frequent complication of both BRVO and CRVO, is a leading cause of RVO-associated vision loss [1]. Because vision loss in BRVO and CRVO is associated with reduced vision-related quality of life [3, 4], ME following RVO represents a considerable public health concern.

Treatment options for RVO-associated ME include grid laser photocoagulation in BRVO, intravitreal corticosteroids, intravitreal antagonists of vascular endothelial growth factor (anti-VEGF), and vitrectomy with internal limiting membrane peeling [5]. Dexamethasone intravitreal implant 0.7 mg (DEX implant, Ozurdex; Allergan plc, Dublin, Ireland) is a biodegradable sustained-release implant that releases dexamethasone into the vitreous over a period of up to 6 months [6]. DEX implant became the first approved medical treatment for RVO-associated ME after phase 3 registration trials conducted at 167 sites in 24 countries worldwide demonstrated its safety and efficacy in improving visual acuity and reducing central retinal thickness (CRT) in patients with BRVO or CRVO [7, 8]. The onset of beneficial effect was rapid, with significant improvement in visual acuity provided by DEX implant 0.7 mg relative to sham procedure within 7 days of treatment [9]. Treatment benefit from a single implant was sustained for several months. The percentage of patients with at least 15 Early Treatment Diabetic Retinopathy Study (ETDRS) letters (three lines) gain in best-corrected visual acuity (BCVA) from baseline was significantly higher in the DEX implant 0.7 mg group than in the sham group at study visits from day 7 to day 90 [7, 9]. For patients who met criteria for re-treatment at month 6 and received a second implant, the second implant demonstrated efficacy and safety similar to the first implant, with the exception of an increase in cataract progression after the second implant [8].

DEX implant was the first treatment reimbursed for RVO-associated ME in France. Following the decision on reimbursement, in November 2010 the French National Authority for Health (Haute Autorité de Santé, HAS) required that a study be implemented to provide information on patterns of DEX implant use and long-term safety and efficacy outcomes associated with DEX implant treatment of RVO-associated ME in French clinical practice. The present study (LOUVRE) was designed and conducted to provide this information.

## Methods

This prospective, multicenter, longitudinal, observational study was conducted at 48 sites in metropolitan France from October 2011 to October 2014. The study protocol was approved by an independent scientific committee and by the French National Authority for Health and the study was conducted in accordance with the ethical standards as laid down in the 1964 Declaration of Helsinki and its later amendments. All patients provided written informed consent prior to study recruitment and enrollment. The study is registered at ClinicalTrials.gov with the identifier NCT01618266.

### Selection of sites

IMS Health (Danbury, CT, USA) identified 150 ophthalmologists who prescribe DEX implant to treat RVO in metropolitan France. After stratification by public versus private practice and by the number of RVO cases treated per month, physicians (75 % private sector, 25 % public sector) were randomly selected, with a principal investigator and no more than one co-investigator selected from any site, and invited to participate in the study. The principal investigators filled out a questionnaire regarding their employment and experience with intravitreal injections.

### Study population

Participating physicians recruited up to 10 consecutive patients who were seen at their sites and met the following screening criteria: at least 18 years of age, and diagnosed with ME following RVO. Patient demographics and ophthalmic histories were collected and ophthalmic examinations were conducted during screening, either on the day of enrollment or at a previous screening visit. The physician decided whether to treat each recruited patient with DEX implant. For patients who were not treated with DEX implant, the reason for not treating was recorded. Patients who were treated with DEX implant were eligible for study enrollment if they resided in metropolitan France and agreed to participate in the study. DEX implant treatment was administered on the day of enrollment (day 0) to all patients who enrolled in the study.

### Visits and assessments

Study visits included the enrollment/DEX implant treatment visit on day 0 (baseline) and follow-up visits scheduled, based on standard practice, at week 6 (±2 weeks), month 4 (±2 weeks), month 6 (±2 weeks), month 12 (±2 months), month 18 (±2 months), and month 24 (±2 months). The time of day for visits was not standardized. At each visit, BCVA was measured in the study eye using an ETDRS chart or the Monoyer scale. Other assessments at each visit included ophthalmic examinations, intraocular pressure (IOP), new treatments and comorbidities since the previous visit, adverse events (AEs) since the previous visit, treatment decisions, and RVO treatments administered. Re-treatment with DEX implant and use of other RVO treatments was at the discretion of the physician and patient. CRT was measured in the 1 mm central macular subfield with optical coherence tomography at screening and each follow-up visit. The NEI VFQ-25 visual function questionnaire [10] was administered at baseline, month 4, and month 24. The French version of this questionnaire has been validated [11] and used in other studies [12, 13].

### Endpoints

The primary endpoint was the change in BCVA from baseline to month 6 in patients treated with DEX implant. Key secondary endpoints included characteristics of patients treated for RVO-related ME with or without DEX implant, and the following endpoints in patients treated with DEX implant: treatment methods; RVO treatments and procedures administered; change in BCVA from baseline at each follow-up visit; percentage of patients with at least 15-letter improvement in BCVA from baseline at each follow-up visit; AEs during the DEX implant injection procedure and throughout follow-up validated by an ophthalmology specialist and coded using MedDRA version 16.1; mean IOP at each visit; and changes in quality of life from baseline to months 4 and 24.

### Statistical analysis

Statistical analysis was performed using SAS version 9.3 (SAS Institute Inc., Cary, NC, USA) and a 2-sided alpha level of 0.05. Categorical variables were analyzed with the Chi square test or Fisher exact test. Changes in BCVA from baseline were analyzed with the Wilcoxon signed-rank test or paired t tests or were estimated from an analysis of covariance (ANCOVA) model. Other continuous variables were analyzed with the Wilcoxon rank-sum test, the Kruskal-Wallis test, or ANCOVA.

Efficacy and safety analyses used observed values with no replacement for missing values in the analysis population of all enrolled patients who had BCVA data available at day 0. In sensitivity analyses, missing data for each visit were replaced with either the median value of the sample or the worst value for the patient during follow-up. Subgroup analyses were based on diagnosis (BRVO vs. CRVO), RVO treatment prior to enrollment (none [treatment-naïve], treated with DEX implant, or treated but not with DEX implant), duration of ME at study enrollment (<3 months or ≥3 months), RVO treatment during follow-up (none, DEX implant alone, or other treatment), and lens status (phakic or pseudophakic).

A sample size of 234 patients was estimated to provide the power to detect a change from baseline BCVA of 1.5 letters at month 6, based on an alpha level of 0.05 and an estimated standard deviation in change from baseline BCVA of 11.7 letters [7]. Assuming 10 % of patients would be lost to follow-up each year and 5 % of patients would have unusable data, enrollment of approximately 300 patients treated with DEX implant was planned to yield an analysis population of 230 patients at month 24.

## Results

### Participating physicians

A total of 76 randomly selected ophthalmologists were invited to participate in the study. Fifty-nine (77.6 %) accepted the invitation, and 48 (63.2 %) recruited at least one patient. The physician questionnaire was completed by 47 of the principal investigators at the 48 active centers. Among the responding physicians, 34 (72.3 %) were male, 25 (53.2 %) were in private practice, eight (17.0 %) worked at a private clinic, one (2.1 %) worked in a private clinic and private practice, eight (17.0 %) worked in a public university hospital, and five (10.6 %) worked in a public general hospital. The physicians had been administering intravitreal injections for a mean of 9 years, and 85.1 % had received training in DEX implant injections.

### DEX implant treatment procedures used

DEX implant injections were given after one or more types of local anesthesia (most commonly eye drops) under aseptic conditions. Injection of DEX implant took place in a dedicated room for 59.2 % of patients and in an operating theater for 40.8 % of patients. Local antibiotics were prescribed for use before injections in 70.9 % of patients, and almost all patients (98.9 %) were prescribed local antibiotics for use after injections.

### Patient recruitment, enrollment, and disposition

**Fig. 1:**
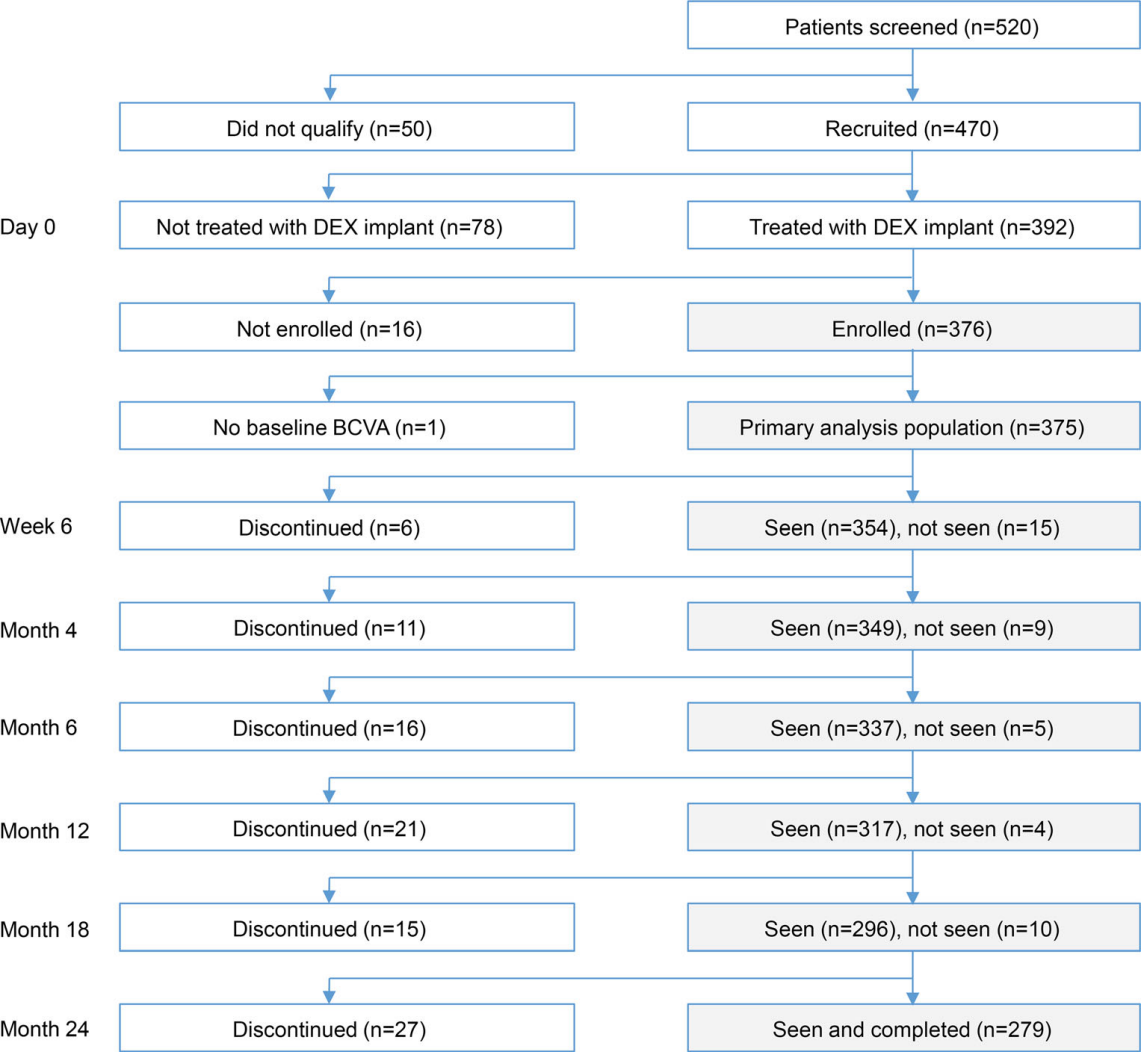
Patient flow through the study. *BCVA* Best-corrected visual acuity, *DEX* implant Dexamethasone intravitreal implant

Patient flow through the study is shown in Fig. 1. A total of 520 patients presented with ME secondary to RVO at the participating sites during the study enrollment period, and 470 (90.4 %) of these patients were recruited for the study. Fifty patients were not recruited because they refused to participate in the study (*n* = 49) or did not live in metropolitan France (*n* = 1). Of the recruited patients, 78 (16.6 %) were not treated with DEX implant for the following reasons: treated with anti-VEGF (*n* = 24), treated with laser (*n* = 7), presented with ischemia (*n* = 13), had glaucoma (*n* = 7), BCVA too low (*n* = 4), BCVA too high (*n* = 3), and other (*n* = 20). Sixteen (4.1 %) patients were treated with DEX implant but were not enrolled, in most cases (*n* = 11) because of patient refusal, and 376 (80.0 %) patients were treated with DEX implant and enrolled in the study. The analysis population consisted of 375 patients, because one enrolled patient had no baseline BCVA or follow-up safety data and was excluded from analysis.

The 2-year study completion rate for the analysis population was 74.4 % (279/375). The most common reason for early patient discontinuation from the study was a protocol violation: follow-up was not possible (*n* = 41, 10.9 %), patient participation in another study (*n* = 1, 0.3 %), or withdrawal of patient consent (*n* = 1, 0.3 %). Other reasons were lack of efficacy (*n* = 23, 6.1 %), patient satisfaction with the treatment (*n* = 10, 2.7 %), death (*n* = 8, 2.1 %), loss to follow-up (*n* = 9, 2.4 %), and treatment-unrelated AE (*n* = 3, 0.8 %).

### Patient characteristics

Demographic and clinical characteristics of recruited and enrolled patients are shown in Table 1. The mean age of patients in the analysis population was 70.3 years, 54.9 % were male, and 53.9 % were diagnosed with BRVO (46.1 % with CRVO). The mean duration of ME at baseline was 10.8 months (Table 1). Mean BCVA was 47.6 ETDRS letters and mean CRT was 554 μm.

**Table 1:** Baseline patient characteristics

	Recruited patients		Enrolled patients
Characteristics	Not treated with DEX implant on day 0 (*N* = 78)	Treated with DEX implant on day 0 (*N* = 392)	Analysis population (*N* = 375)
Age, mean (SD), years	72.7 (11.4)	70.4 (11.2)	70.3 (11.2)
Range	34–90	29–93	29–93
Gender, *n* (%)			
Male	43 (55.1)	214 (54.6)	206 (54.9)
Female	35 (44.9)	178 (45.4)	169 (45.1)
Diagnosis in study eye, *n* (%)			
BRVO	47 (60.3)	211 (53.8)	202 (53.9)
CRVO	31 (39.7)	181 (46.2)	173 (46.1)
Time since onset of ME, mean (SD), months	16.6 (26.3)	11.3 (18.5)	10.8 (18.1)
Median	6.1	3.5	3.3
BCVA in study eye, mean (SD), ETDRS letters	47.0 (25.6)	48.2 (21.4)	47.6 (21.2)
Range	0–85	0–85	0–85
CRT in study eye, mean (SD), μm	482 (204)	551 (179)	554 (180)
Ischemia status of the RVO, *n* (%)			
Ischemic	15 (19.7)	33 (8.5)	32 (8.6)
Non-ischemic	45 (59.2)	218 (56.0)	209 (56.2)
Mixed	16 (21.1)	138 (35.5)	131 (35.2)
Previous treatment for RVO, *n* (%)^a^			
No (treatment-naïve)	27 (34.6)	153 (39.2)	145 (38.9)
Yes (previously treated)	51 (65.4)	239 (61.0)	228 (61.1)
Local ophthalmic treatment			195 (52.3)
DEX implant	23 (29.5)	153 (39.2)	149 (39.9)
Laser photocoagulation	29 (37.2)	116 (29.6)	112 (30.0)
Triamcinolone acetonide	6 (7.7)	51 (13.0)	49 (13.1)
Bevacizumab	23 (29.5)	37 (9.4)	34 (9.1)
Ranibizumab	20 (25.6)	25 (6.4)	24 (6.4)
Systemic treatment^b^	23 (29.5)	114 (29.1)	111 (29.8)
Lens status in the study eye, *n* (%)^c^			
Phakic	55 (70.5)	284 (72.4)	273 (72.8)^d^
Pseudophakic	23 (29.5)	107 (27.3)	101 (26.9)
Ocular comorbidities in study eye, *n* (%)			
Glaucoma	25 (32.1)	53 (13.5)	52 (13.9)
Ocular hypertension	26 (33.3)	75 (19.1)	73 (19.5)
Age-related macular degeneration	3 (3.8)	6 (1.5)	5 (1.3)
Cataract	18 (23.1)	110 (28.1)	108 (28.8)
IOP in study eye, mean (SD), mm Hg	17.0 (7.1)	14.6 (3.4)	14.7 (3.0)

*BCVA* Best-corrected visual acuity, *BRVO* Branch retinal vein occlusion, *CRT* Central retinal thickness, *CRVO* Central retinal vein occlusion, *DEX implant* Dexamethasone intravitreal implant 0.7 mg, *ETDRS* Early Treatment Diabetic Retinopathy Study, *IOP* Intraocular pressure, *ME* Macular edema, *RVO* Retinal vein occlusion, *SD* Standard deviation

^a^Percentages in analysis population based on *N* = 373 because two patients had missing data

^b^Systemic RVO treatments included aspirin, troxerutin, hemodilution, and other

^c^Data missing for one eye

^d^Among the phakic eyes, 165 were without cataract and 108 were with cataract

Most patients (61.1 %) in the analysis population had been previously treated for RVO: 52.3 % had received local ophthalmic treatment, most commonly DEX implant (39.9 %), laser (30.0 %), or anti-VEGF (15.5 %), and 29.8 % of patients had received systemic treatment (Table 1). Overall, 38.9 % of patients in the analysis population were treatment naïve, 39.9 % had been treated previously with DEX implant, and 21.2 % had been treated previously for RVO, but not with DEX implant (Table 1).

Most patients (56 %, 209/375) in the analysis population had a history of or current cataract; 101 of these patients had undergone cataract surgery and 108 were not yet operated. The other most common comorbidities were hypertension (19.5 %) and glaucoma (13.9 %). In comparison with patients treated with DEX implant, patients not treated with DEX implant had higher mean IOP and thinner CRT, and were more likely to have ischemic RVO and less likely to have been previously treated with DEX implant (Table 1).

### Treatments during the study (analysis population)

Patients were administered a mean of 2.6 DEX implant injections during the 2-year study. All patients were treated with DEX implant at baseline, and the majority were re-treated during follow-up. During the study, 124 patients (33.1 %) received only one implant (at baseline), 92 (29.3 %) received two implants, 59 (18.8 %) received three implants, 43 (13.7 %) received four implants, 38 (12.1 %) received five implants, 16 (5.1 %) received six implants, and three (1.0 %) received seven implants. The mean (±SD) time between DEX implant injections for patients who received multiple injections was 6.6 ± 3.6 months.

At the time of the 6-month primary efficacy endpoint, 254 patients (67.7 %) had received only DEX implant for treatment of RVO during the study, and 121 patients (32.3 %) had received both DEX implant and other types of RVO treatment. By month 24, a total of 167 patients (44.5 %) had received only DEX implant for treatment of RVO during the study period, including 61 patients (16.3 %) who received only the baseline DEX implant injection and no RVO treatments during follow-up, and 106 patients (28.3 %) who were re-treated with DEX implant and received a mean (±SD) of 2.4 ± 1.3 DEX implant injections (range, 1–6) after the baseline treatment. The remaining 208 patients (55.5 %) received treatment other than DEX implant for RVO during the study period, including 63 patients (16.8 %) who received a mean of 3.1 ± 2.0 recorded treatments with other RVO therapies (range, 1–10) and no DEX implant during follow-up, and 145 patients (38.7 %) who received DEX implant (mean of 2.3 ± 1.4 DEX implant treatments, range 1–6) and moved to other RVO therapy (mean of 2.5 ± 1.6 recorded treatments with other therapies, range 1–7) during follow-up.

For the 208 patients who used other RVO therapy after one or more DEX implant treatments, the first use of other therapy occurred at a mean of 8.7 ± 6.4 months, with most use of other RVO therapy occurring in the second year of the study. The other RVO therapies used were usually local ophthalmic treatments, namely laser (106 patients, 28.3 %), ranibizumab (114 patients, 30.4 %), bevacizumab (12 patients, 3.2 %), and aflibercept (five patients, 1.3 %). Among the 114 patients who received ranibizumab, the first injection was at or after month 12 in 71.1 %, at or after month 18 in 60.8 %, and at month 24 in 46.4 %.

For patients who were administered other RVO treatment, the reason given was usually the recurrence of macular edema (36 % of cases) or the presence of ischemia (27 % of cases). Physicians reported no reason for the switch in therapy in 22 % of cases and other reasons in 15 % of cases.

### Efficacy outcomes (analysis population)

**Fig. 2:**
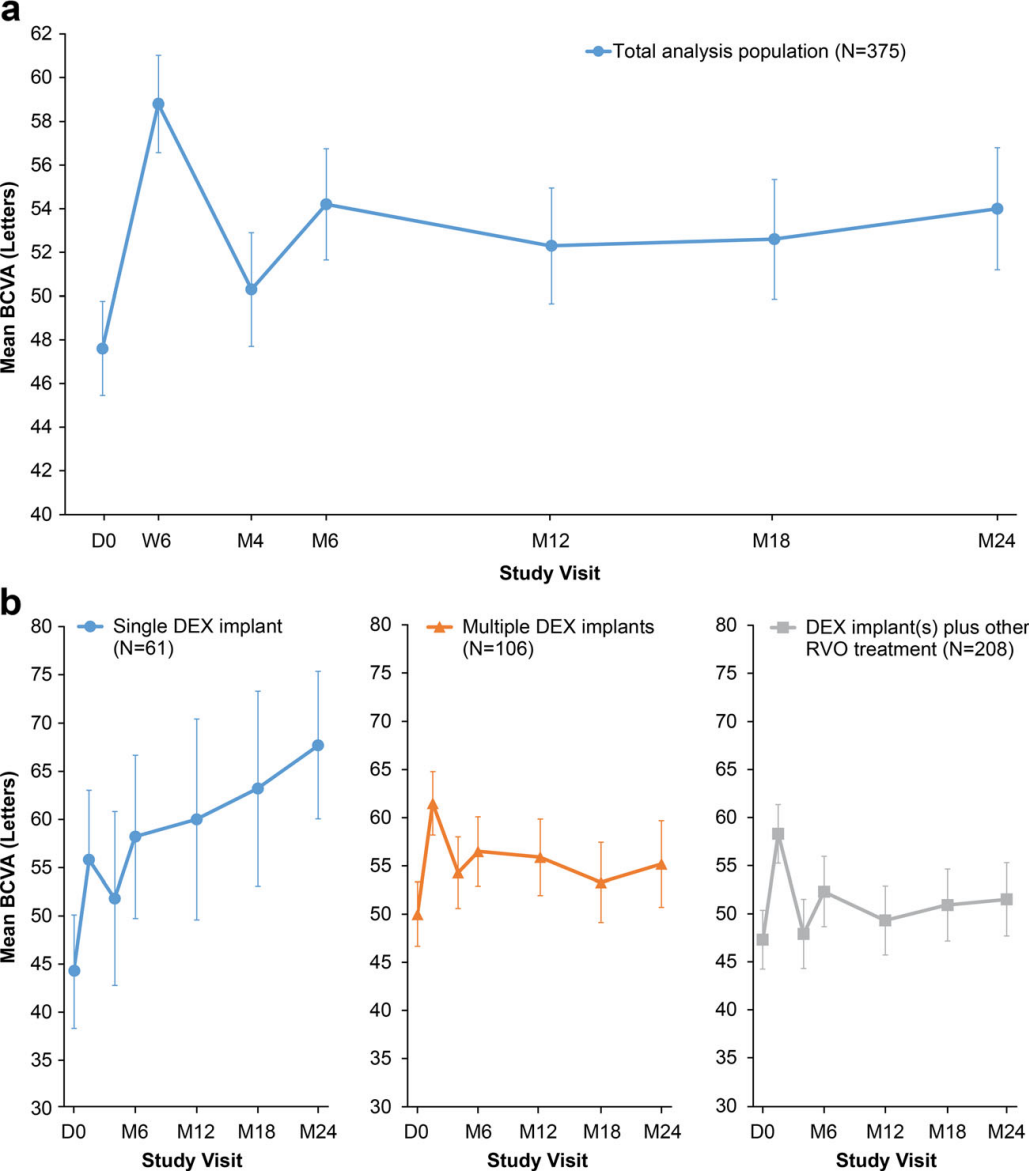
Mean BCVA in study eye. **a** Total analysis population. **b** Subgroups based on all RVO treatments received during the study. Error bars show 95 % confidence interval. *BCVA* best-corrected visual acuity, *D* day, *DEX implant* dexamethasone intravitreal implant, *M* month, *RVO* retinal vein occlusion, *W* week

Mean BCVA in the total analysis population had increased from baseline by 11.4 ± 16.4 letters (*p* < 0.001) at the first follow-up visit (week 6), when the peak value was reached (Fig. 2a). The improvement in BCVA from baseline continued to be statistically significant at all subsequent follow-up visits (*p* ≤ 0.014). The mean (SD) change in BCVA from baseline was 5.1 (19.0) letters (*p* < 0.001, Wilcoxon signed rank test) at month 6 (primary efficacy endpoint), and the gain in BCVA seen at month 6 was sustained through month 24. At month 24, the mean (SD) change in BCVA from baseline was 4.6 (22.3) letters (*p* < 0.001). The percentage of patients with ≥15-letter gain in BCVA from baseline was 43.4 % at week 6, 31.3 % at month 6, and 38.7 % at month 24.

The analysis of the primary endpoint used data from 323 patients, as there were 52 patients with missing BCVA data at month 6. However, results were similar in sensitivity analyses with missing values imputed. Moreover, the primary endpoint at month 6 was significant both in patients who had received only DEX implant treatment and in patients who had received additional other RVO treatment within the first 6 months of the study; the mean (SD) change from baseline BCVA at month 6 in these cohorts was 5.5 (18.8) letters (*p* < 0.001, *N* = 254) and 4.2 (19.6) letters (*p* = 0.006, *N* = 121), respectively. The primary endpoint was also significant in patients who received a single DEX implant and no other RVO treatment at visits up to and including the month 6 visit; the mean change in BCVA from baseline to month 6 for these patients was +5.3 letters (*p* = 0.011, *N* = 91).

Analysis of BCVA in subgroups defined by the treatment received during the 24-month study—a single DEX implant, ≥2 DEX implants, or DEX implant and other RVO treatments—showed BCVA gain by week 6 in each subgroup (Fig. 2b). The improvement in BCVA from baseline (Table 2) was significant in each subgroup at month 6 (*p* ≤ 0.006, ANCOVA). In patients treated with DEX implant only, BCVA continued to improve after month 6; the mean change from baseline BCVA at month 24 was +8.3 (95 % CI: 4.4, 12.2) letters (*p* < 0.0001, ANCOVA). BCVA change from baseline at month 24 was +20.7 (95 % CI: 12.4, 29.0) letters in the subgroup treated with a single DEX implant (*p* < 0.001, ANCOVA) and +4.9 (95 % CI: 0.5, 9.2) letters in the subgroup treated with ≥2 DEX implants (*p* = 0.029, ANCOVA). In the subgroup that received DEX implant and other RVO treatment, BCVA was fairly stable after month 6, and the mean change from baseline BCVA at month 24 was +2.3 (95 % CI: −0.8, 5.5) letters (*p* = 0.143, ANCOVA). The mean (SD) time of the initial “other” treatment in this subgroup was at 8.7 (6.4) months, suggesting that moving to other treatment did not result in improved BCVA in this cohort.

**Table 2:** Key efficacy outcomes in total analysis population and subgroups

Population	Mean (SD) baseline BCVA, letters	Mean (SD) change from baseline BCVA at 6 months, letters	Mean (SD) change from baseline BCVA at 24 months, letters	Patients with ≥15-letter gain from baseline BCVA at 6 months, %	Patients with ≥15-letter gain from baseline BCVA at 24 months, %
Total analysis population (*N* = 375)	47.6 (21.2)	5.1 (19.0)	4.6 (22.3)	31.3	38.7
Subgroups: diagnosis					
BRVO (*N* = 202)	52.8 (17.4)	5.5 (19.6)	4.8 (20.5)	32.4	39.9
CRVO (*N* = 173)	41.5 (23.5)	4.6 (18.4)	4.4 (24.2)	30.0	37.4
Subgroups: duration of ME at baseline^a^					
<3 months (*N* = 180)	43.3 (22.8)	8.5 (20.5)	6.7 (26.3)	40.3	47.9
≥3 months (*N* = 183)	51.4 (19.0)	2.0 (17.5)	3.0 (18.5)	23.0	30.1
Subgroups: lens status at baseline^b^					
Pseudophakic (*N* = 101)	50.2 (19.4)	4.2 (20.7)	3.0 (22.2)	30.2	36.2
Phakic without cataract (*N* = 165)	48.9 (20.9)	7.9 (17.0)	7.1 (21.3)	36.2	45.2
Phakic with cataract (*N* = 108)	43.3 (22.8)	1.4 (20.0)	2.1 (23.9)	24.2	30.4
Subgroups: treatment status at baseline					
Naïve (*N* = 145)	43.9 (23.1)	7.5 (20.0)	5.9 (26.7)	35.3	45.7
Prior DEX treatment (*N* = 149)	52.5 (18.9)	2.0 (16.0)	2.1 (17.6)	22.3	29.7
Prior treatment, not DEX (*N* = 79)	45.3 (20.2)	8.4 (21.1)	7.6 (23.1)	43.8	45.2
Subgroups: RVO treatment during study					
Through 6 months^c^					
DEX only (*N* = 254)	49.3 (19.2)	6.1 (1.2)		29.9	
Single DEX treatment (*N* = 91)	47.8 (21.3)	6.1 (2.3)		29.7	
≥2 DEX treatments (*N* = 163)	50.1 (18.0)	6.0 (1.5)		29.9	
≥1 DEX and other RVO treatments (*N* = 121)	44.0 (24.6)	3.0 (1.8)		34.3	
Through entire 24 months^c^					
DEX only (*N* = 167)	47.9 (19.7)	7.0 (1.6)	8.3 (2.0)	30.9	45.3
Single DEX treatment (*N* = 61)	44.3 (23.0)	9.3 (3.1)	20.7 (4.2)	37.1	73.9
≥2 DEX treatments (*N* = 106)	50.0 (17.3)	6.2 (1.8)	4.9 (2.2)	28.7	37.3
≥1 DEX and other RVO treatments (*N* = 208)	47.3 (22.4)	3.7 (1.3)	2.3 (1.6)	31.6	34.5

*BCVA* Best-corrected visual acuity, *BRVO* Branch retinal vein occlusion, *CRVO* Central retinal vein occlusion, *DEX implant* Dexamethasone intravitreal implant, *ME* Macular edema, *RVO* Retinal vein occlusion, *SD* Standard deviation

^a^Duration of ME data missing for 12 patients

^b^Lens status data missing for one patient

^c^Mean (SD) change from baseline values are from an ANCOVA model using baseline BCVA as the covariate

Analyses of BCVA in subgroups defined by diagnosis, duration of ME, and previous treatment also showed significant gains in BCVA at month 6 in each subgroup (Table 2). At baseline, mean BCVA was worse in patients with CRVO compared with patients with BRVO (41.5 vs. 52.8 letters, *p* < 0.001). BCVA improved significantly from baseline in both subgroups at 6 and 24 months (*p* < 0.001), with no significant difference in BCVA gain between patients with BRVO and patients with CRVO. At month 6, BCVA had improved from baseline in patients with recent-onset (<3 months) (*p* < 0.001) and persistent (≥3 months) (*p* = 0.013) ME. However, the mean gain in BCVA was greater in patients with recent-onset ME (8.5 vs. 2.0 letters, *p* < 0.001). Mean BCVA at baseline was higher in patients with a duration of ME ≥3 months (51.4 vs. 43.3 letters, *p* < 0.001), but in an ANCOVA model that adjusted for baseline BCVA, a similar between-group difference in BCVA change from baseline was seen, suggesting that patients with recent-onset ME responded better to treatment. BCVA also improved significantly from baseline at 6 months in patients regardless of their treatment status at baseline (treatment-naïve, *p* < 0.001; previously treated with DEX implant, *p* = 0.032; and previously treated without DEX implant, *p* < 0.001). However, there was a significant difference among subgroups in the percentage of patients gaining at least 15 letters in BCVA from baseline at both 6 months and 24 months, with patients previously treated with DEX implant less likely to gain at least 15 letters (Table 2). This result may be explained in part by the higher mean BCVA at baseline in patients previously treated with DEX implant (Table 2).

Among the 375 patients in the analysis population, study eyes in 101 (26.9 %) had already undergone cataract surgery and were pseudophakic at enrollment, and cataract was present at enrollment (based on AE reports) in 108 of the 273 phakic study eyes (39.5 %) (data on lens status were missing for 1 eye). BCVA gains were seen in both phakic and pseudophakic eyes, although as might be expected, gains were smaller in phakic eyes with cataract than in those without cataract (Table 2). At month 6, the mean improvement in BCVA from baseline was statistically significant in pseudophakic eyes (+4.2 letters, *p* = 0.010) and phakic eyes without baseline cataract (+7.9 letters, *p* < 0.001), but not in phakic eyes with baseline cataract (+1.4 letters, *p* = 0.272) (Table 2).

**Fig. 3:**
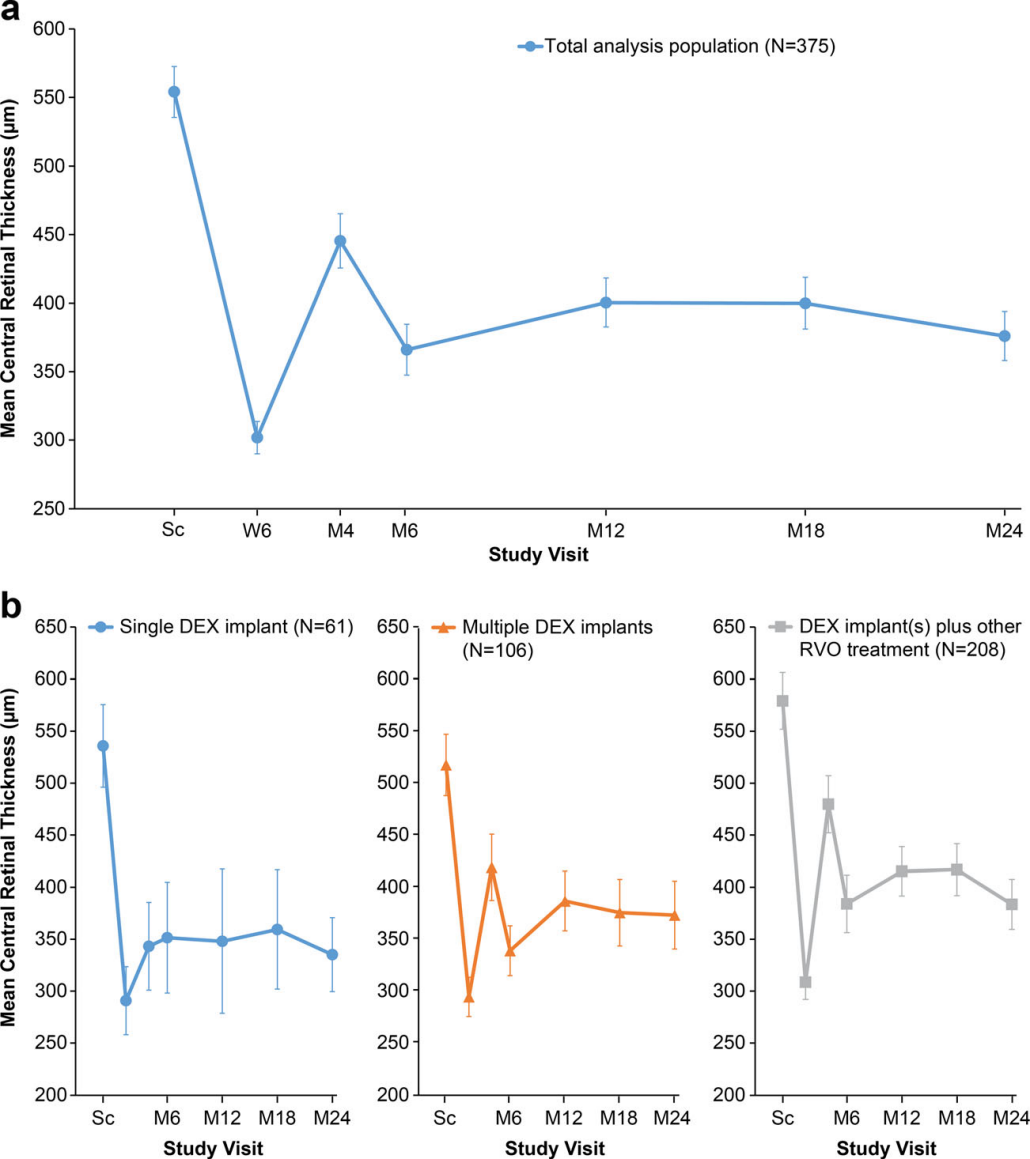
Mean CRT in study eye. **a** Total analysis population. **b** Subgroups based on all RVO treatments received during the study. Error bars show 95 % confidence interval. *CRT* central retinal thickness, *DEX implant* dexamethasone intravitreal implant, *M* month, *RVO* retinal vein occlusion, *Sc* screening, *W* week

Mean reductions in CRT from the screening value were statistically significant at each follow-up visit in the total analysis population and in each subgroup defined by treatment received over 24 months (single DEX implant, ≥2 DEX implants, and DEX implant and other RVO treatment) (*p* < 0.001, Wilcoxon rank signed test). Peak improvement in CRT was seen at week 6, with mean (±SD) CRT in the total analysis population decreasing from 554 ± 180 μm at screening to 302 ± 111 μm at week 6 (Fig. 3). Mean CRT was stable from month 6 to month 24 in the total population and each subgroup (Fig. 3). At month 4, the reduction in CRT was larger in the subgroup that received a single DEX implant for RVO treatment than in the other subgroups (*p* < 0.001).

### Safety outcomes (analysis population)

AEs were reported in 261 (69.6 %) patients, and treatment-related AEs were reported in 162 (43.8 %) patients. The incidence of AEs differed among the subgroups defined by treatment pattern (*p* < 0.001). The incidence of AEs was 47.5 % (29/61) in patients treated with a single DEX implant, 69.8 % (74/106) in patients treated and re-treated with DEX implant, and 76.0 % (158/208) in patients treated with DEX implant plus other RVO treatment, consistent with the total number of RVO treatments administered in these subgroups (mean of 1, 2.4, and 4.3 treatments, respectively). The most common AEs were IOP increase and cataract. IOP increase was reported as an AE in 129 patients (34.4 %), and in 99 of these patients (26.4 %), the IOP increase was considered to be treatment related. Glaucoma was reported as an AE in 12 patients (3.2 %), and in four of these patients (1.1 %), the glaucoma was considered to be potentially related to treatment. One of these patients underwent trabeculoplasty between months 12 and 18, and the AE was reported to be resolved at month 18. Glaucoma in the other patients was managed with IOP-lowering medication. There were no incisional glaucoma surgeries during the study period. Cataract was present at enrollment in 108 of the phakic patients (39.6 %). During the study, cataract-related AEs (cataract, subcapsular cataract, nuclear cataract, cortical cataract, or cataract surgery) were reported in 149 patients (54.6 % of phakic patients), and in 82 of these patients, the AEs were considered to be potentially related to treatment. One hundred and ten patients (40.1 % of phakic patients) underwent cataract surgery. These patients were not excluded from the analyses of BCVA. There were no reports of endophthalmitis during the study.

**Fig. 4:**
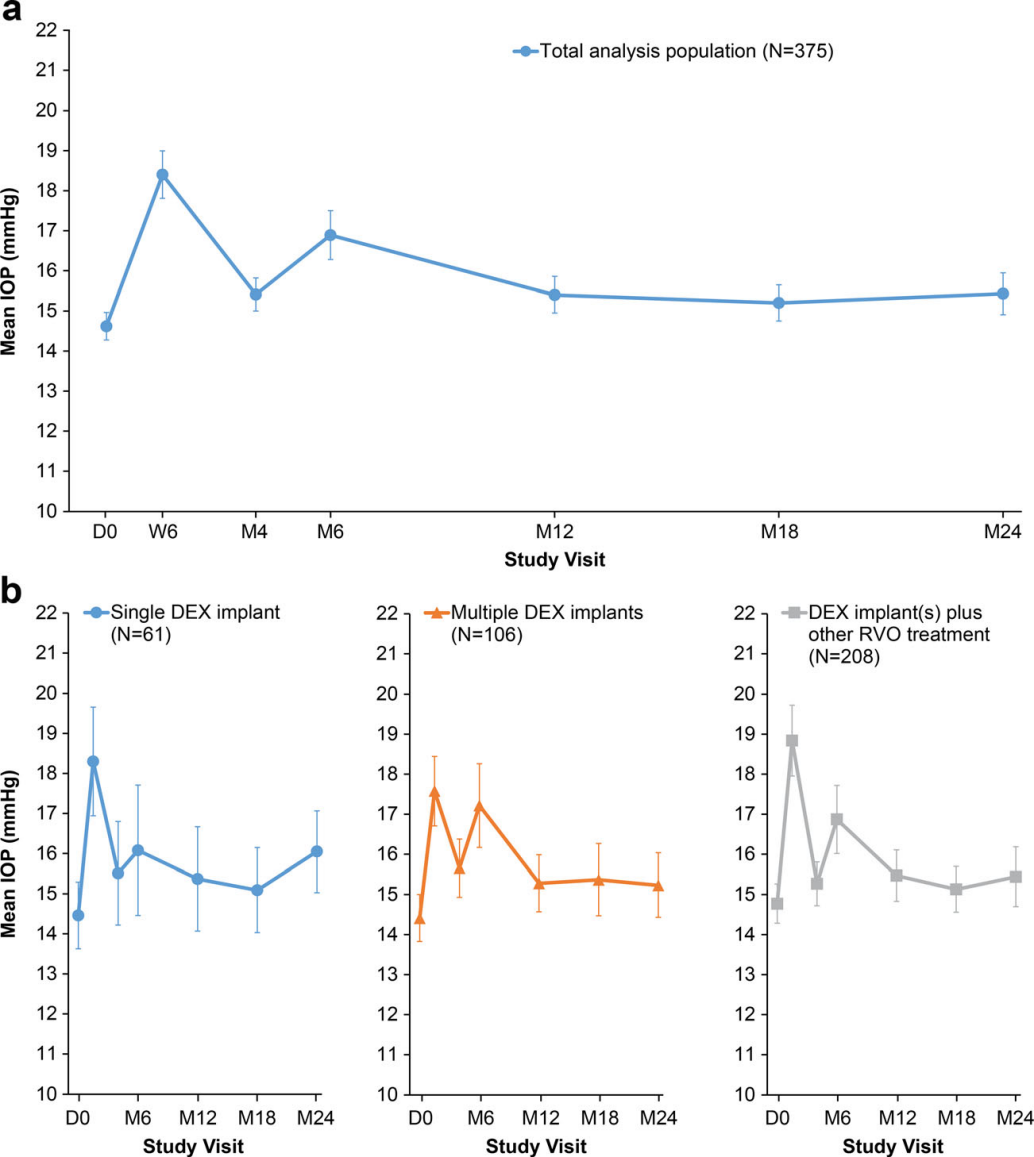
Mean IOP in study eye. **a** Total analysis population. **b** Subgroups based on all RVO treatments received during the study. Error bars show 95 % confidence interval. *D* day, *DEX implant* dexamethasone intravitreal implant, *IOP* intraocular pressure, *M* month, *RVO* retinal vein occlusion, *W* week

Mean IOP in the analysis population was highest at week 6 (Fig. 4a), when mean IOP had increased to 18.4 mmHg from the baseline value of 14.6 mmHg. At study visits during the second year, mean IOP was near baseline levels (Fig. 4a). Mean IOP in the subgroups defined by treatment pattern did not differ significantly among subgroups at any study visit (Fig. 4b). Overall, the mean (±SD) change in IOP during the study was +1.4 (±4.9) mmHg. For the majority of patients (74.1 %, 254/343), any increase in IOP during the study was ≤5 mmHg, whereas 25.9 % (89/343) of patients had an increase in IOP of >5 mmHg from baseline. Only one patient had an IOP measurement >30 mm Hg at any time during the study.

IOP-lowering medication was initiated in 82 patients (21.9 %) at week 6 after the initial DEX implant treatment. Use of IOP-lowering medication in these patients was reduced to 43 patients (11.5 %) at month 6 and 21 patients (5.6 %) at month 24.

### Quality of life

In the total analysis population, the overall mean VFQ-25 score improved from 75.9 at baseline to 77.9 at month 4 and 78.7 at month 24. There was no significant difference in overall mean VFQ-25 scores among the subgroups defined by treatment pattern over 24 months, but patients treated with a single DEX implant only or with ≥2 DEX implants only showed better improvement in VFQ-25 scores than patients treated with DEX implant and other RVO treatments (mean change in scores of +3.9, +2.6 and +1.8, respectively; *p* < 0.001 among groups).

## Discussion

This prospective, observational study demonstrated the efficacy and safety of DEX implant for treatment of RVO-associated ME in the French clinical setting. With a mean of only 2.6 injections over 2 years, DEX implant provided clinically significant improvement in BCVA and CRT at month 6 that was sustained through month 24. For 44.5 % of patients, DEX implant was the only RVO treatment administered during the 24-month period. The improvement in BCVA was associated with improved vision-related quality of life in treated patients. The side effect profile of DEX implant treatment was as expected and consistent with previous reports of DEX implant treatment in patients with RVO [8]. The most common adverse effects were increases in IOP and cataract progression, which were manageable.

Various patterns of use of DEX implant in the French clinical setting were identified in the study. Among the 167 patients who received only DEX implant for treatment of their ME during the study, 61 patients received only a single DEX implant during the 2-year study period. These patients had significantly better reduction of CRT at month 4 than patients who received additional DEX implant treatments or other types of RVO therapy, and the level of CRT reduction was maintained through the remainder of the study, suggesting that these patients received only a single DEX implant because of the success of the treatment. The remaining 106 patients who received no RVO treatments other than DEX implant during the study received a mean of 3.4 DEX implants over 2 years, and 37.3 % of these patients had achieved at least 15-letter improvement in BCVA from baseline at month 24.

A total of 208 patients received other RVO treatment, in addition to DEX implant, at some point during the 24-month study. The other RVO treatment was usually administered after the primary endpoint at month 6. During the course of the study, ranibizumab was approved and reimbursed for treatment of RVO-associated ME in France, and this explains why a substantial number (122) of the patients who used other RVO treatment during the study were switched to ranibizumab treatment. There was no clear benefit in moving to other RVO treatment, as gains in BCVA and improvement in CRT were similar in patients who were re-treated with DEX implant only and those who used other RVO treatment. Furthermore, the incidence of AEs was higher in patients who used both DEX implant and other RVO treatments than in patients who used only DEX implant. However, a selection bias may have contributed to these latter results, as patients who were treated with both DEX implant and other RVO treatments may have represented more complicated or relapsing cases.

In other subgroup analyses, DEX implant demonstrated efficacy in improving BCVA in subgroups defined by diagnosis, the duration of ME at baseline, previous treatment for ME, and lens status. Improvements in BCVA were similar between patients with BRVO and patients with CRVO at 6 and 24 months. In the GENEVA study, DEX implant also demonstrated efficacy in both CRVO and BRVO, but the improvement in BCVA was not sustained through 6 months in patients with CRVO [7]. In this study, DEX implant treatment significantly improved BCVA regardless of the duration of ME at baseline, but gains were greater in patients with recent-onset ME (<3 months). These findings are consistent with the GENEVA study results showing that clinically significant gains in BCVA and improvement in CRT following DEX implant treatment were more likely in patients with RVO of shorter duration, particularly for patients with BRVO [14]. The results confirm the benefit of early treatment of RVO-associated ME with DEX implant and suggest that DEX implant is a good first-line treatment for patients with RVO-associated ME. The disadvantage of DEX implant use is the occurrence of AEs associated with intraocular corticosteroid therapy (increases in IOP and cataract).

In subgroup analysis based on previous treatment, mean increases in BCVA from baseline in treatment-naïve patients were large (7.5 and 5.9 letters at 6 and 24 months, respectively), as expected given the relatively shorter duration of ME in these patients. In patients previously treated with other RVO treatments, substantial mean increases in BCVA from baseline (8.4 and 7.6 letters, respectively) also were seen at months 6 and 24, suggesting that patients treated with other types of RVO therapy may benefit from a switch to DEX implant. Patients previously treated with DEX implant had better BCVA at enrollment; in these patients, the increase in BCVA from baseline at month 6 was smaller, but still significant, suggesting that BCVA improvement provided by previous DEX implant treatment was maintained after DEX implant treatment during the study. In the total analysis population, the mean increase in BCVA at 6 months (5.1 letters) was similar to that seen in the GENEVA registration study (4.6 to 5.5 letters, Allergan data on file). In the DEX implant 0.7 mg treatment group of the GENEVA study, the percentage of patients who were treatment-naïve was higher (>90 %) and the mean duration of ME at baseline was shorter (~5 months) than in the LOUVRE study, so better efficacy of DEX implant might be expected. However, 155 (41.3 %) patients in LOUVRE received a second implant before month 6, and this might be expected to improve outcomes at 6 months.

Retrospective observational studies of DEX implant in the treatment of RVO-associated ME in the clinical setting have generally reported a slightly shorter interval between DEX implant injections. In a retrospective study in Germany, the mean time between DEX implant treatments in 87 RVO patients who received at least two treatments was 5 months (4.5 months in CRVO patients and 5.5 months in BRVO patients) [15]. At a mean of 2.75 months after the last DEX implant injection, the mean gain in BCVA from baseline was nine ETDRS letters, and improvement in BCVA was greater in patients with a duration of ME <90 days than in the total patient population, consistent with the findings in LOUVRE. In a retrospective study in 289 RVO patients who received multiple injections of DEX implant in the United States (SHASTA), the mean interval between injections was 5.6 months, and 30 to 35 % of patients gained at least 15 letters in ETDRS BCVA from baseline after each of the first five injections [16]. Consistent with the findings in LOUVRE, efficacy outcomes were similar in patients treated with DEX implant only and patients treated with DEX implant plus other RVO therapy [17].

This study has several limitations. Comparison of outcomes between patients treated with DEX implant alone and those moved to other RVO treatments could be limited by patient selection bias if the patients moved to other RVO treatments differed in disease characteristics. Other RVO treatments were allowed, and it is difficult to determine to what extent those treatments affected patient outcomes. Because participating physicians did not always record other RVO treatments at non-study visits, the number of anti-VEGF treatments in the cohort who received other RVO treatments is probably underestimated. Ocular AEs were not reported separately for study eyes and fellow eyes, so the incidence of cataract in study eyes could have been overestimated. The rate of 24-month study completion was only 74.4 %, in large part because it was impossible to continue follow-up for 10.9 % of patients. A high rate of discontinuations is expected in long-term observational studies but can bias the results if patients who discontinue and patients who remain in the study differ in their response to treatment.

In summary, DEX implant treatment of RVO-associated ME was associated with good visual and anatomic outcomes in the French clinical setting. The effects of DEX implant are long lasting. Treatment benefit was achieved with a mean of less than three injections over 2 years, with re-treatment given when needed at a mean interval of 6.6 months. Outcomes were most favorable when RVO-associated ME was treated soon after onset. Patients who switched from DEX implant to other RVO treatments (most commonly laser and ranibizumab treatment) did not have improved outcomes in this study.
